# Improving Indel Detection Specificity of the Ion Torrent PGM Benchtop Sequencer

**DOI:** 10.1371/journal.pone.0045798

**Published:** 2012-09-19

**Authors:** Zhen Xuan Yeo, Maurice Chan, Yoon Sim Yap, Peter Ang, Steve Rozen, Ann Siew Gek Lee

**Affiliations:** 1 Division of Medical Sciences, National Cancer Centre Singapore, Singapore, Singapore; 2 Department of Medical Oncology, National Cancer Centre Singapore, Singapore, Singapore; 3 Oncocare Cancer Centre, Gleneagles Medical Centre, Singapore, Singapore; 4 Neuroscience and Behavioral Disorders, Duke-NUS Graduate Medical School, Singapore, Singapore; Leuven University, Belgium

## Abstract

The emergence of benchtop sequencers has made clinical genetic testing using next-generation sequencing more feasible. Ion Torrent's PGM^TM^ is one such benchtop sequencer that shows clinical promise in detecting single nucleotide variations (SNVs) and microindel variations (indels). However, the large number of false positive indels caused by the high frequency of homopolymer sequencing errors has impeded PGM^TM^'s usage for clinical genetic testing. An extensive analysis of PGM^TM^ data from the sequencing reads of the well-characterized genome of the *Escherichia coli* DH10B strain and sequences of the *BRCA1* and *BRCA2* genes from six germline samples was done. Three commonly used variant detection tools, SAMtools, Dindel, and GATK's Unified Genotyper, all had substantial false positive rates for indels. By incorporating filters on two major measures we could dramatically improve false positive rates without sacrificing sensitivity. The two measures were: B-Allele Frequency (BAF) and VARiation of the Width of gaps and inserts (VARW) per indel position. A BAF threshold applied to indels detected by UnifiedGenotyper removed ∼99% of the indel errors detected in both the DH10B and *BRCA* sequences. The optimum BAF threshold for *BRCA* sequences was determined by requiring 100% detection sensitivity and minimum false discovery rate, using variants detected from Sanger sequencing as reference. This resulted in 15 indel errors remaining, of which 7 indel errors were removed by selecting a VARW threshold of zero. VARW specific errors increased in frequency with higher read depth in the *BRCA* datasets, suggesting that homopolymer-associated indel errors cannot be reduced by increasing the depth of coverage. Thus, using a VARW threshold is likely to be important in reducing indel errors from data with higher coverage. In conclusion, BAF and VARW thresholds provide simple and effective filtering criteria that can improve the specificity of indel detection in PGM^TM^ data without compromising sensitivity.

## Introduction

Recent years have witnessed a rapid increase in the utilization of next-generation sequencing (NGS) technology for clinical genetic testing [1]–[6]. In particular, mutation screening by the resequencing of bacterial, viral and cancer genomes from clinical samples have resulted in the discovery of disease-associated genetic factors [7]–[10].

Even more recently, benchtop high-throughput sequencers such as the Ion Torrent PGM^TM^ from Life Technologies and the MiSeq from Illumina have emerged as the latest options for genome resequencing [11], [12]. Their lower start-up costs and simpler sample preparation promise to reduce the reliance on core genome facilities and are likely to spur the use of NGS in clinical genetic testing.

The Ion Torrent PGM^TM^ is a commercially available benchtop high-throughput sequencer capable of analysing clinically derived genomes with high productivity and performance [5], [13]. However, the PGM^TM^ produces high frequencies of homopolymer sequencing errors [14].

**Figure 1:**
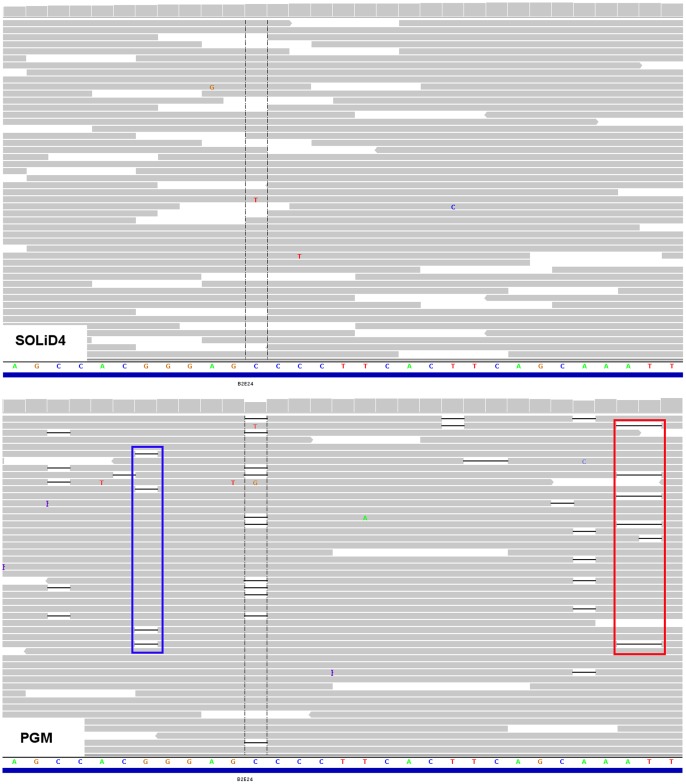
Comparison of read-pileups derived from SOLiD and Ion Torrent sequencing at a homopolymer region. Both top and bottom panels were IGV pileups derived from the SOLiD4 and Ion Torrent sequencing of exon 24 of the *BRCA2* gene respectively. At the interval marked by two dotted lines, a deletion was detected in the PGM^TM^ data. This deletion was not detected in SOLiD4 data. In addition, spurious gaps were observed in other sequence positions in the PGM^TM^ data whereas none of these gaps were seen in the SOLiD4 data. Many of these spurious gaps were aligned to sequence positions which were followed by a run of the same nucleotide. The region highlighted by the red rectangular box indicates a false positive deletion that was associated with a non-zero VARW and the blue rectangular box indicates a false positive deletion that was associated with a low BAF.

Homopolymer sequencing errors are those associated with runs of consecutive identical nucleotides (Fig. 1). These errors tend to increase in genomic regions where the occurrence of true polymorphisms is also higher [15], and thus it is analytically challenging to reduce these errors without compromising detection sensitivity.

High sensitivity and specificity in screening genomic variations are essential for clinical genetic tests. Any excessive errors may have significant impact on verification time and costs; and most importantly, treatment decisions might be affected.

To correct for homopolymer sequencing errors, resequencing the region of interest many times or using alternative platforms for verification have been used [16], [17]. Although this approach can improve detection specificity, it is impractical for clinical laboratories due to time and cost constraints, especially when dealing with a high volume of samples. Several other studies [18]–[20] have considered ignoring variants detected at regions with longer homopolymer run length. While this reduced the false positive rate, some true positives were discarded, resulting in lower sensitivity.

Alternatively, error modelling approaches have been employed during variant calling. SAMtools [21], Dindel [15] and GATK’s UnifiedGenotyper [22], are common variant callers that attempt to incorporate the prediction of homopolymer indel errors in their algorithms. However, to our knowledge, there have been no reports of analyses of the performance of these variant callers using PGM^TM^ data.

In this study, we show that there are a large number of false positive indels called using SAMtools, Dindel and GATK’s UnifiedGenotyper, from the DH10B genome and germline human *BRCA1* and *BRCA2* sequences. Subsequently, we show that by using a simple two-step filtering approach with B-Allele Frequency (BAF) and VARiation of the Width of gaps and inserts (VARW), about 99% of the homopolymer indel errors were eliminated without compromising detection sensitivity.

## Results

### Excessive false positive indels in PGM^TM^ data for the DH10B genome

The DH10B genome has been resequenced on multiple platforms, and any variants detected are likely to be false positives.

Alignment was generated using proprietary software, CASAVA, Bioscope, and Torrent Suite, as well as an open source aligner, BWA. DH10B sequence data generated on the PGM^TM^ and aligned with the Torrent Suite had zero SNVs and 42 indels detected using SAMtools (Table 1). Dindel generated zero SNVs and 478 indels, while there were two SNVs and 204 indels detected using GATK's UnifiedGenotyper. Overall, when the same data was aligned using BWA, fewer indels were called (Table 1).

**Table 1:** Variants in DH10B detected by different platforms and filter settings.

Sequencer (Variant caller)	Aligner/Workflow	SNVs	Indels	Aligner/Workflow	SNVs	Indels
MiSeq (UnifiedGenotyper)	CASAVA	0**	1**	BWA	0**	1**
SOLiD4 (UnifiedGenotyper)	Bioscope	0**	0	BWA	9	2
PGM (SAMtools)	Torrent Suite	0	42	BWA	0	24
PGM (Dindel)	Torrent Suite	0	478	BWA	0	314
PGM (UnifiedGenotyper)	Torrent Suite	2	204	BWA	0	144
PGM (UnifiedGenotyper + Filtered*)	Torrent Suite	0	1	BWA	0	1

* Filtered using BAF and VARW thresholds.

** Expected number of SNVs and indels.

The results were compared to variants detected using MiSeq and SOLiD4 which were not expected to have a significant number of homopolymer sequencing errors. The MiSeq data had no SNVs and one indel; while the SOLiD data had no SNVs or indels when using the BAM file generated by Bioscope. However, when BWA was used to align the SOLiD4 data, additional false positives were called (Table 1). Manual inspection using Integrated Genomics Viewer (IGV) [23] indicated that the detected indel from the MiSeq data was likely to be a true novel variant (Fig. S1).

### Characteristics of BAF and VARW for false positive indels from PGM^TM^ data detected in the DH10B genome

**Figure 2:**
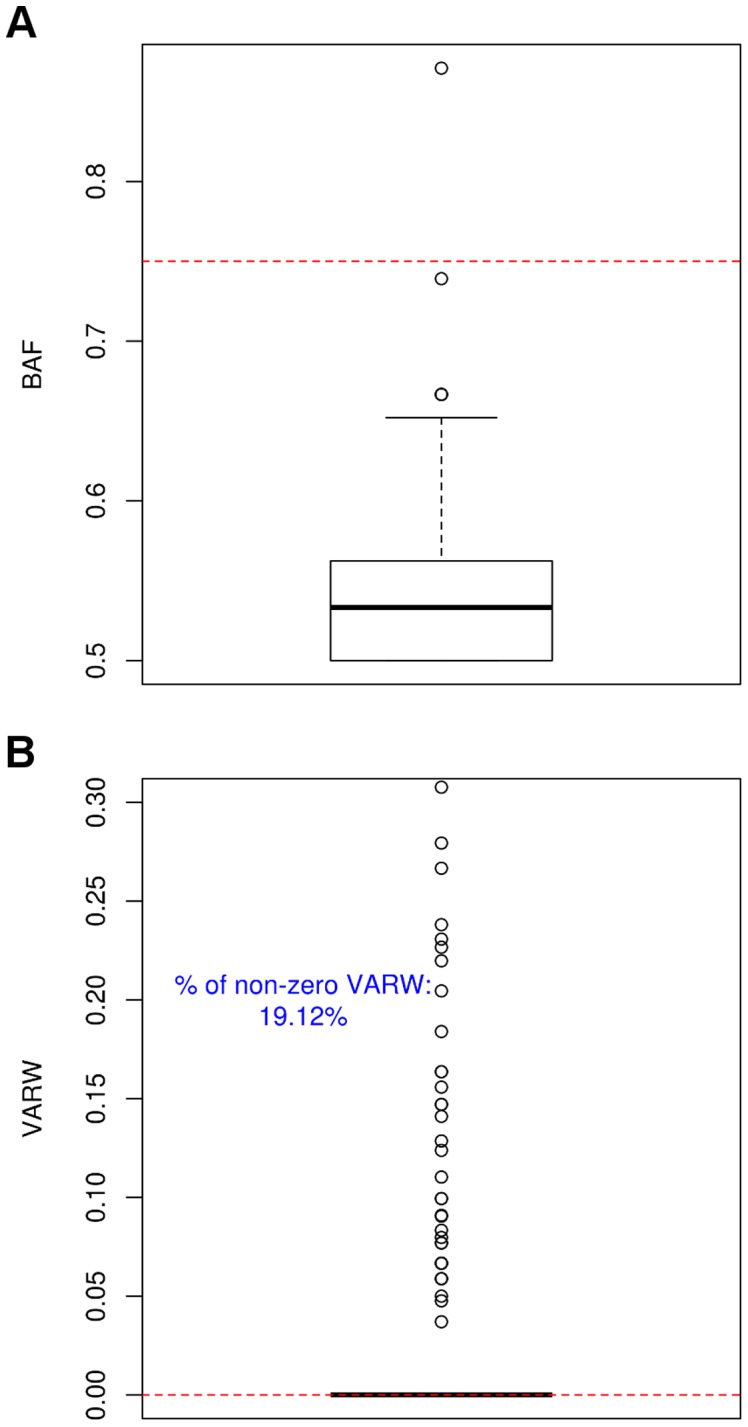
Distribution of BAFs and VARWs for indels derived from DH10B. Boxplots for BAFs and VARWs of indels detected from PGM^TM^ data were plotted. (A) The majority of BAFs were skewed towards the minimum. The dotted red line indicates the selected BAF_th_ of 0.75. Only one indel exceeded this BAF_th_. (B) The majority of the VARWs were equal to zero, indicated by a dotted red line. The percentage represents the proportion of non-zero VARW.

Haploid call correction was applied to remove all indels with BAFs of <0.5 detected using UnifiedGenotyper. The distribution of BAFs for all the remaining indels is shown in Fig. 2A. The majority of BAFs derived from DH10B's false positive indels were skewed towards lower values, where more than 75% were below 0.6 albeit the DH10B is a haploid genome. Since there was only one true indel, the distribution of BAFs shown in Fig. 2A could be considered as the distribution of false positive indels.

Ideally, a true indel would be signaled by reads containing gaps or inserts of uniform width, i.e. a VARW of zero. Any deviation from this criterion suggests a potential error. In the DH10B sequence, 19.12% of indels had non-zero VARW (Fig. 2B).

Approximately 74% of false positive indels with non-zero VARWs were associated with homopolymer run length >2 bases (Fig. S2). In contrast, only about 35% of false positive indels with zero VARWs were associated with homopolymer run length >2 bases.

### Filtering threshold for removing indels and SNVs from DH10B

A BAF value of 0.75 was selected as the threshold (BAF_th_) for filtering (Fig. 2A) so that the number of indels from PGM^TM^ data did not exceed the amount detected using either MiSeq or SOLiD4. All non-zero VARW associated indels were removed using a BAF_th_ of 0.75. After BAF filtering of the PGM^TM^ data, there was a single indel remaining, which was the same indel detected using MiSeq. (Fig. S1). We believe this is a true variant.

### Excessive false positive indels in PGM^TM^ data for *BRCA1* and *BRCA2* sequences

We analyzed *BRCA1* and *BRCA2* sequences derived from six patients, with the patients' samples subjected to Sanger, SOLiD4, and PGM^TM^ sequencing. The *BRCA* genes have multiple regions with homopolymer runs, making them ideal candidates for our study. SNVs and indels identified by Sanger sequencing were considered to be true variants, and there were in total 33 SNVs and 3 indels detected.

An abundance of false positive indels were detected in the PGM^TM^ data using both SAMtools (n = 64) and UnifiedGenotyper (n = 2000) (Table 2). SAMtools only managed to detect two of the three ‘true’ indels and generated more false negative SNVs (n = 17). Although no false negatives were detected by UnifiedGenotyper, there were 20 false positive SNVs and 2000 false positive indels extracted from the *BRCA* sequences.

**Table 2:** Variants in *BRCA1* and *BRCA2* detected by different platforms and filter settings.

		SNVs				Indels			
Sequencer (Variant caller)	Aligner/Workflow	FP	FN	TP	TN	FP	FN	TP	TN
SOLiD4 (UnifiedGenotyper)	CLC	16	0	33	148744	2	0	3	148788
SOLiD4 (UnifiedGenotyper)	BWA	16	0	33	148744	4	0	3	148786
PGM (SAMtools)	BWA	24	17	16	148736	64	1	2	148726
PGM (UnifiedGenotyper)	BWA	20	0	33	148740	2000	0	3	146790
PGM (UnifiedGenotyper + Filtered*)	BWA	17	0	33	148743	8	0	3	148782

Only ‘callable’ base were considered which were the sum of all bases in coding exons with ≥4X read coverage from the six samples (n = 148793).

FP = False positives; FN = False negatives; TP = True positives; TN = True negatives, as determined by Sanger sequencing.

* SNVs were filtered using BAF_th_ = 0.2. Indels were filtered using BAF_th_ = 0.28 and VARW_th_ = 0.

When BWA was used to align the SOLiD4 data, there were only minor differences in the number of called variants when compared to the numbers called from BAM files generated by the CLC Genomics Workbench.

### Characteristics of BAF and VARW for false positive indels from PGM^TM^ data in *BRCA1* and *BRCA2* sequences

**Figure 3:**
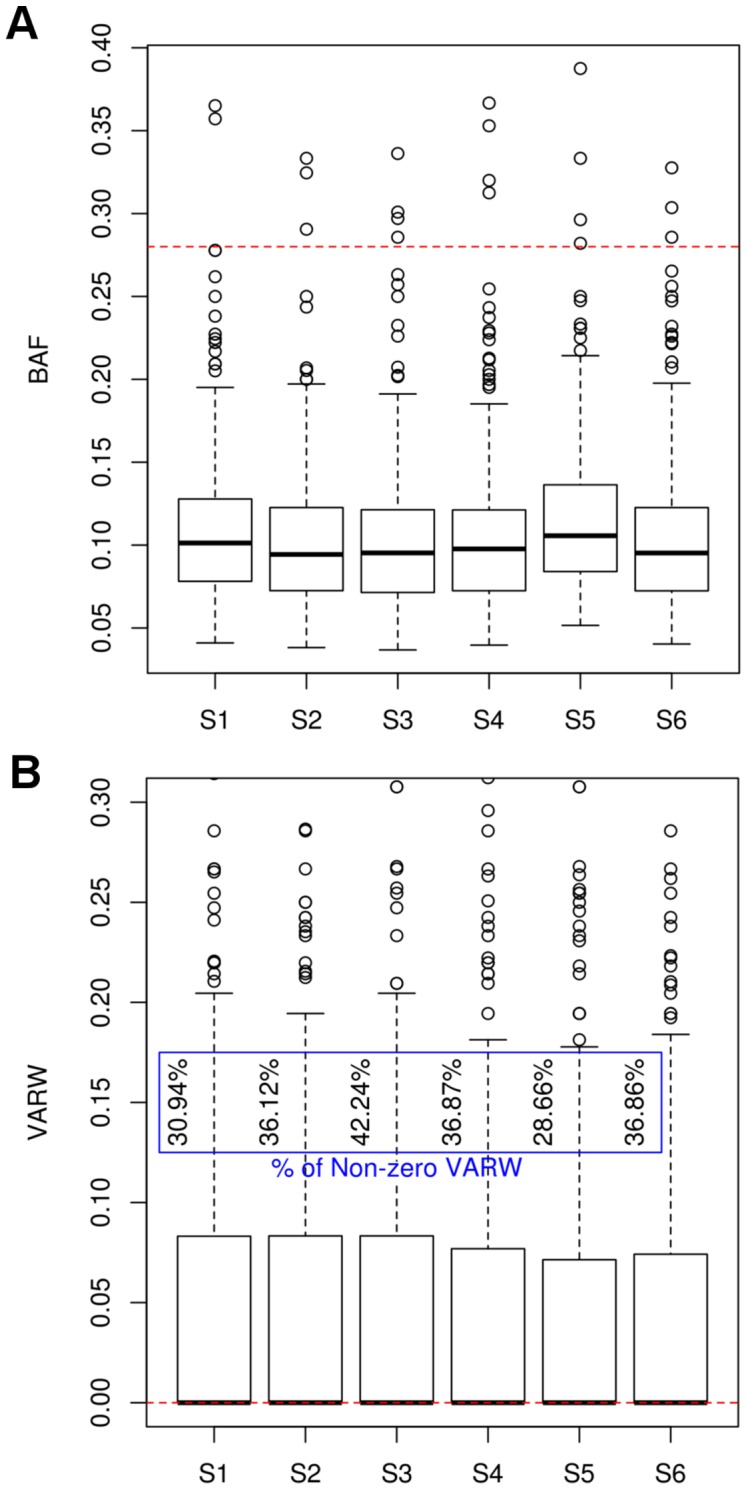
Distribution of BAFs and VARWs for indels derived from the *BRCA1* and *BRCA2* sequences of six patients. Boxplots for BAFs and VARWs of indels detected from PGM^TM^ data of six patients were plotted. (A) The majority of BAFs were skewed towards the minimum. The dotted red line indicates the selected BAF_th_ of 0.28. (B) The majority of the VARWs were equal to zero, indicated by a dotted red line. The percentages represent the proportion of non-zero VARWs.

To investigate the characteristics of BAFs and VARWs for variants from *BRCA1* and *BRCA2* sequences, we focused on indels detected using UnifiedGenotyper. BAFs of indels for *BRCA1* and *BRCA2* tended to be low in each sample (Fig. 3A) with a mean of 0.1 and a standard deviation of 0.004. The maximum BAFs of the six samples had an average of 0.1967, much less than the theoretical value of 0.5 for a heterozygous allele. Across the six samples, between 28% and 43% of the called indels had non-zero VARW (Fig. 3B). Since the number of ‘true’ indels (n = 3) was much less than the number of called indels (n = 2000), the distributions in Fig. 3 approximate the distributions of false positive indels.

### Selection and performance of BAF and VARW thresholds for the *BRCA1* and *BRCA2* data

The BAF thresholds for SNVs and indels for BRCA sequences were selected based on the criteria of 100% sensitivity and minimum False Discovery Rate (FDR), for variants detected using UnifiedGenotyper. For SNVs, a BAF_th_ of 0.2 was selected, with a FDR of 0.34. For indels, we selected a VARW_th_ of zero to filter all indels with non-zero VARW. The optimum BAF_th_ for indels was determined to be 0.28, to achieve 100% sensitivity and a minimum FDR of 0.73. Seven false positive indels were removed by using a VARW_th_ of zero.

Applying BAF_th_ and VARW_th_ (Table 2, Fig. S3) resulted in a decrease in the number of false positive SNVs and indels. For SNV detection, the number of false positives was reduced from 20 to 17 (Table 2). More significantly, there was an average 250-fold reduction in the total number of false positive indels (Table 2). All of the variants detected by SOLiD and Sanger sequencing remained after applying the filters (Table 2).

### Read depth and the number of false positive indels from *BRCA1* and *BRCA2* sequences

**Figure 4:**
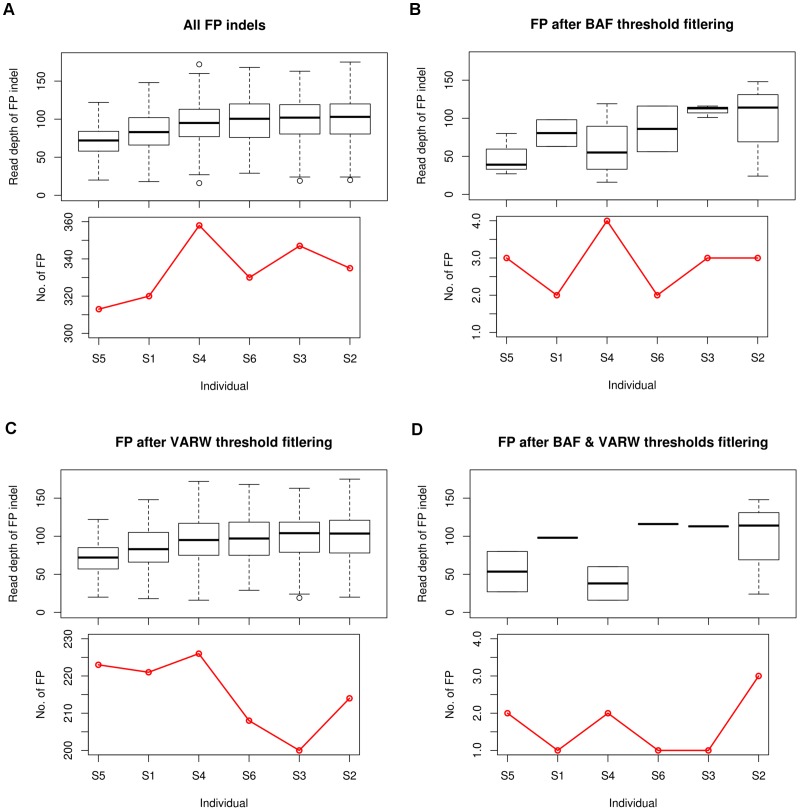
Number of false positive indels from *BRCA1* and *BRCA2* sequencing of six samples with varying read depth. (A–D) (Top) Read depth distribution of false positive (FP) indels in each sample; (Bottom) number of false positive indels in each sample. (A) Number of FP indels before BAF and VARW filtering. (B) Number of FP indels after BAF filtering. (C) Number of FP indels after VARW filtering. (D) Number of FP indels after BAF and VARW filtering.

For indels called by UnifiedGenotyper, we observed that increased read depth does not necessarily reduce the number of false positive indels (Fig. 4). More false positive indels with high read depth (≥90X) were detected in samples S2, S3, S4 and S6 (Fig. 4A). After filtering with BAF_th_ or both BAF_th_ and VARW_th_, no obvious trend was observed between read depth and the remaining number of false positives (Fig. 4B, 4D). Interestingly, applying VARW_th_ reduced more false positives in samples with ≥100X median read depth at indel sites (Fig. 4C).

## Discussion

Homopolymer-associated indel errors are a known challenge for variant detection using NGS technologies [14], [15], [24]–[26]. In mutation screening of patients' samples, the high false positive rate of such indel errors has hindered its clinical application due to the strict requirement of detection sensitivity and specificity. We investigated the characteristics of homopolymer associated indel errors derived from one such technology, the Ion Torrent PGM^TM^, and suggest a solution to reduce the false positive rate for indels. Based on the different pipelines studied, the best sensitivity and specificity were achieved with BWA, GATK's UnifiedGenotyper and the BAF_th_ and VARW_th_ filters. The use of BWA generally helped to reduce the number of false positive SNVs and indels detected from MiSeq and PGM^TM^ reads for the DH10B dataset. However, for the DH10B dataset from SOLiD4 sequencing, more false positives were extracted using BWA, which may suggest that the aligner is not optimized for color space data. Although GATK generated a higher number of false positives than SAMtools, SAMtools appeared to have lower sensitivity (Table 2).

The different characteristics of indel errors under various homopolymer contexts (varying homopolymer run length) were illustrated previously by Albers et al using data generated from the Illumina platform [15]. However, our data suggests that homopolymer indel errors generated by PGM^TM^ behave differently from those generated by Illumina such that they do not necessarily follow a ‘1/f’ BAF distribution as highlighted by Albers et al [15] (Fig. 3A and Fig. S5).

For *BRCA* sequencing using PGM^TM^, a substantial amount of false positive indels were removed from the output of UnifiedGenotyper with a BAF_th_ of 0.28. However, further reduction of the false positive rate by applying a larger BAF_th_ resulted in reduced sensitivity.

We observed that the false positive indels that were not removed by BAF_th_ tended to be in sequences with longer homopolymer runs (Fig. S2, S4). Moreover, the reads at these indel positions contain gaps and inserts that are varying in width (Fig. 1, 2B, 3C). This observation led us to consider the addition of the VARW filter, which removed a further 7 false positive indels. Notably, applying the VARW threshold did not affect the detection sensitivity, which was 100% for the *BRCA* sequences (Table 2).

Each *BRCA* sequence had a mean read depth that ranged between 50X and 100X and this was sufficient to obtain a 100% variant calling sensitivity. Increasing sequencing depth is usually expected to reduce error rates during variant discovery since most of the random errors within reads are not expected to generate sufficient BAF. However, our results showed that the number of false positives tended to be higher in data with higher median read depths, suggesting that the falsely generated inserts and gaps were not distributed randomly. This non-randomness is likely due to the bias associated with homopolymer regions. The addition of VARW_th_ filtering appears to be helpful in reducing the read depth dependent indel errors (Fig. 4C). With the current sequencing depths, we detected SNVs and indels with allele frequencies as low as 0.2.

To demonstrate the SNV calling accuracy of PGM^TM^, the number of detected SNVs before and after BAF_th_ filtering was determined (Tables 1 and 2). For the DH10B dataset, the BAF_th_ filter removed all false positive SNVs detected by UnifiedGenotyper. However, relatively few false positive SNVs called by UnifiedGenotyper were removed from the *BRCA* sequencing dataset (n = 3/20) and the improvement in specificity was much lower than that for indels. Thus, a simple BAF_th_ does not characterize the majority of SNV errors extracted from PGM^TM^ data. Nevertheless, in contrast to indel calling, the number of false positive SNVs detected using PGM^TM^ was comparable to that obtained from other next generation sequencing platforms.

Lastly, the BAF filtering relied on the correct estimation of BAF_th_. For excluding errors from *BRCA* sequences, we set the criteria of 100% sensitivity and minimum FDR to select the BAF_th_ and VARW_th_ filters, which can be easily applied to homogeneous DNA samples, such as that extracted from peripheral blood of patients. The estimation of BAF_th_ can become complicated in DNA samples derived from a mixed population of cells, for instance in tumor samples. Nevertheless, alternative approaches to estimate BAF_th_ have been proposed previously to deal with tumor data [27], [28]. In addition, the BAFs of the sequenced regions are non-uniform even in homogeneous DNA samples. DNA contamination, PCR bias and strand bias are potential sources that have an impact on the BAF at specific loci. Such locus-specific BAF can also limit the use of a simple threshold. We anticipate that the better estimation of BAF_th_ is critical and likely to improve the performance of variant detection from PGM^TM^ data.

Similar homopolymer indel errors have been observed using the Roche 454 sequencer [25]. To reduce such errors, AmpliconNoise [29] and Denoiser [30] are commonly employed. AmpliconNoise infers the read sequences from pyrosequencing flowgrams using a likelihood approach, where error-free data is used to train parameters. Denoiser implements a rank-abundance curve model to cluster reads. A novel tool, Acacia, has recently been described that uses a statistical approach to identify errors by comparing raw signals against signals from known homopolymer over- and under-calls [31]. However, all three approaches have a varying error reduction percentage ranging from 6%–90% [31]. In addition, all of these approaches remove or correct errors during base-calling which is computationally intensive as it requires the processing of a large amount of raw signals. Furthermore, these approaches are less accessible since many investigators generally obtain their data in the form of read sequences from sequencing service providers. In this current study, the BAF_th_ and VARW_th_ filters reduced indel errors in PGM^TM^ data by 99% during variant calling. Although untested with Roche 454 data, these filters could potentially be useful in reducing homopolymer indel errors called by the Roche 454 sequencer.

In conclusion, we have showed that current variant callers generated large numbers of false positive indels from PGM^TM^ data. Low BAF was identified as the major property of the indel errors generated by PGM^TM^, and can be used to exclude false positive indels. At genomic sites with long homopolymer runs and covered with more reads, filtering of variants with a non-zero VARW removed additional indel errors in this study.

## Materials and Methods

### Identification of genomic variants from the *Escherichia coli* DH10B genome

FASTQ files and BAM files (aligned with proprietary Torrent Suite or CASAVA workflows) were downloaded from Life Technologies' Ion Community (http://lifetech-it.hosted.jivesoftware.com/login.jspa) and Illumina's Scientific Data for MiSeq Personal Sequencer (http://www.illumina.com/systems/miseq/scientific_data.ilmn). For the SOLiD4 data, CSFASTQ, QUAL and BAM files generated using the proprietary Bioscope workflow were obtained from Applied Biosystems' SOLiD4 system DH10B data repository (http://solidsoftwaretools.com/gf/project/dh10b2×50/). The Ion Torrent's single-end data was generated using an Ion 316 chip on the Ion Torrent PGM^TM^.

In order to investigate the impact of aligners on variant calling, additional BAM files were generated by aligning FASTQ reads (or CSFASTQ/QUAL reads for SOLiD4) using BWA 0.9.2. All mapped MiSeq reads with ≥6 mismatches were removed.

An implementation of the GATK variant discovery workflow (version 1.3–24-gc8b1c92) [22] was applied to the BAM file from each platform to detect SNVs and indels. Briefly, BAM files were preprocessed by local realignment and deduplication, and the SNVs and indels were called using GATK's UnifiedGenotyper and VariantFiltration. Variants derived from regions of low mapping quality, low read depth (<4X) and strong strand bias were removed. GATK, SAMtools and Dindel were originally designed for the detection of variants in diploid genome. For the haploid DH10B genome, a 'haploid call correction' was applied such that variants with BAF of less than 0.5 were not considered in the subsequent analyses.

### Identification of genomic variants from *BRCA1* and *BRCA2*

#### Preparation of DNA samples

Peripheral blood and buccal wash samples were obtained from six patients attending outpatient clinics at the National Cancer Centre Singapore. Written informed consent was obtained from all contributing volunteers, and ethics approval for this study was obtained from the Centralized Institutional Review Board of SingHealth (Singapore). DNA was extracted using an optimized in-house method [32]. DNA concentration and purity were assessed using Nanodrop 1000 (Nanodrop Technologies, Wilmington, DE), and all DNA samples had A260/280 nm ratios between 1.8 and 2.0.

#### Generation of Sanger sequencing data and variant detection

PCR amplification and thermal cycling were carried out on complete coding regions of *BRCA1* and *BRCA2*, including about 40 bp of non-coding regions flanking the 5' and 3' ends of each exon as described (manuscript in preparation). Cycle sequencing was carried out using BigDye Terminator kit version 3.1 (Applied Biosystems) according to manufacturer’s instructions. Sequencing products were analyzed on a 3100 Genetic Analyzer (Applied Biosystems). Sequence alignment and variant detection were done using DNASTAR 8.0 SeqMan Pro.

#### SOLiD sequencing

Pooled PCR amplicons generated for the *BRCA1* and *BRCA2* genes, containing about 1ug DNA per sample were sequenced using the ABI SOLiD 4 platform (Applied Biosystems) through a service provider, 1st BASE Pte Ltd (Singapore). Fragment libraries were constructed using the strategy of concatenation of PCR amplicons, end-repair, and physical shearing, followed by the manufacturer’s protocol. Agarose gel electrophoresis was used to confirm the mean size of the fragments (150–175 bp). Barcoding, nick translation, and emulsion PCR was carried out according to the standard manufacturer's protocol in the SOLiD™ Fragment Library Barcoding Kit (Applied Biosystems). Each sample, containing pooled PCR amplicons from one patient, was represented by a distinct barcode. Sequencing was carried out on one-quad of a slide on the ABI SOLiD 4 platform.

#### Ion Torrent PGM^TM^ sequencing

NGS data from the PGM^TM^ was generated from 100 ng of pooled PCR amplicons for the *BRCA1* and *BRCA2* genes. Fragment libraries were constructed using the strategy of DNA fragmentation, barcode and adaptor ligation, library amplification, and fragment size selection using agarose gel electrophoresis. The target fragment size distribution is 180–220 bp for the PGM^TM^. Fragment libraries were constructed according to the manufacturer's instructions as stipulated in the Ion DNA Barcoding kit (Life Technologies). DNA was recovered from the agarose gel using PureLink Gel Extraction kit (Life Technologies). Size distribution of DNA fragments were analysed on the Agilent Bioanalyzer using the High Sensitivity Kit (Agilent, Santa Clara, CA). Template preparation and emulsion PCR, and Ion Sphere Particles (ISP) enrichment was done using the Ion Xpress Template kit (Life Technologies) according to the manufacturer's instructions. The quality of the resultant ISPs was assessed using Qubit 2.0 Fluorometer (Life Technologies), and were loaded and sequenced on a 316 chip (Life Technologies).

#### Mapping assembly and variant discovery from NGS data

The SOLiD raw reads underwent adapter trimming, removal of reads shorter than 20 bp and removal of exact duplicates, as well as quality trimming. The pre-processed reads were aligned using CLC Genomics Workbench 4.03 against reference sequences corresponding to the *BRCA1* and *BRCA2* genes (refseq NG_005905.2 and NG_012772.1). This was followed by SNV and indel detection. Each variant within the exonic regions of *BRCA1* and *BRCA2* was verified against the Sanger sequencing data.

For each of the six samples, the PGM^TM^ raw reads were aligned against the human reference genome (1000 genome project build 37) using BWA 0.9.2. To detect variants, a GATK workflow similar to that used for DH10B variant discovery, was applied to the aligned data with additional base quality score recalibration. All detected variants within the coding exons of *BRCA1* (RefSeq ID: NG_005905.2) and *BRCA2* (RefSeq ID: NG_012772.1) were considered for subsequent analyses.

### Calculation of BAF and VARW

BAF was derived from the frequency of reference and variant alleles specified in the Variant Calling Format (VCF) file generated from GATK variant discovery workflow:
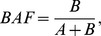
where:


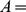
reference allele frequency.


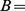
non-reference allele frequency.

For each detected indel, VARW was calculated by finding the variance of the width of inserted/deleted sequences:
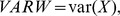
where:


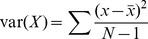
, 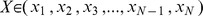



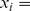
 the width of gap/insert in one out of 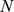 reads that covered a given indel position, 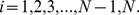


## Supporting Information

Figure S1 **A potential novel insertion detected in DH10B.** The top, middle and bottom are IGV pileups at a selected region generated by data from MiSeq, filtered PGM^TM^, and SOLiD 4 respectively. The selected region showed a potential novel insertion that was not found in the DH10B genome (highlighted by dotted lines). The insertion was not detected in SOLiD4 possibly because the position of the insertion was mainly covered at the end of reads.
(TIF)

Figure S2 **Homopolymer run lengths of indels filtered and unfiltered by VARW threshold in DH10B.** Panels (A) and (B) are barplots showing the proportions of indels with different homopolymer run lengths in filtered and unfiltered data derived from the *E.coli* DH10B strain respectively. BAF_th_ was set to zero so that only the effect of VARW was evaluated. (A) More than 74% of the filtered indels were found to be associated with homopolymer run length >2 bases; (B) In contrast, only about 34.55% were mapped to the same homopolymer run length profile.
(TIF)

Figure S3 **Comparison of false positive detection before and after filtering analysis.** Boxplots comparing the number of false positive SNVs and indels from unfiltered and filtered results detected in the *BRCA1* and *BRCA2* sequences of 6 samples were plotted in (A) and (B) respectively. (A) There were less than 5 false positive SNVs detected in each of the 6 samples. (B) The red numeric numbers indicate the mean number of false positive indels before and after filtering with BAF_th_ and VARW_th_.
(TIF)

Figure S4 **Homopolymer run length of indel errors removed only by VARW in** ***BRCA*** **sequences.** Barplot showing the proportions of indels with different homopolymer run lengths in filtered data derived from *BRCA* sequences. All filtered indels were associated with homopolymer run length >3.
(TIF)

Figure S5 **Indel BAF spectrum at different homopolymer contexts derived from DH10B and** ***BRCA*** **sequences.** (A) Shows the indel spectrum derived from DH10B. (B) Shows the corresponding spectrum for the *BRCA* genes for samples S3, S5 and S6. The 'HRun' refers to the homopolymer run length which indicates that the spectrum was plotted under a given run length (homopolymer context). Samples S3, S5 and S6 were shown to have 3 indels in total by SOLiD and Sanger resequencing. The density shifted toward higher BAF values along with the increase of HRun in both (A) and (B).
(TIF)
