# Supplementary figures and images for: Improving Indel Detection Specificity of the Ion Torrent PGM Benchtop Sequencer
Source: PLoS One. 2012 Sep 19;7(9):e45798. doi: 10.1371/journal.pone.0045798 (PMC3446914; doi:10.1371/journal.pone.0045798)

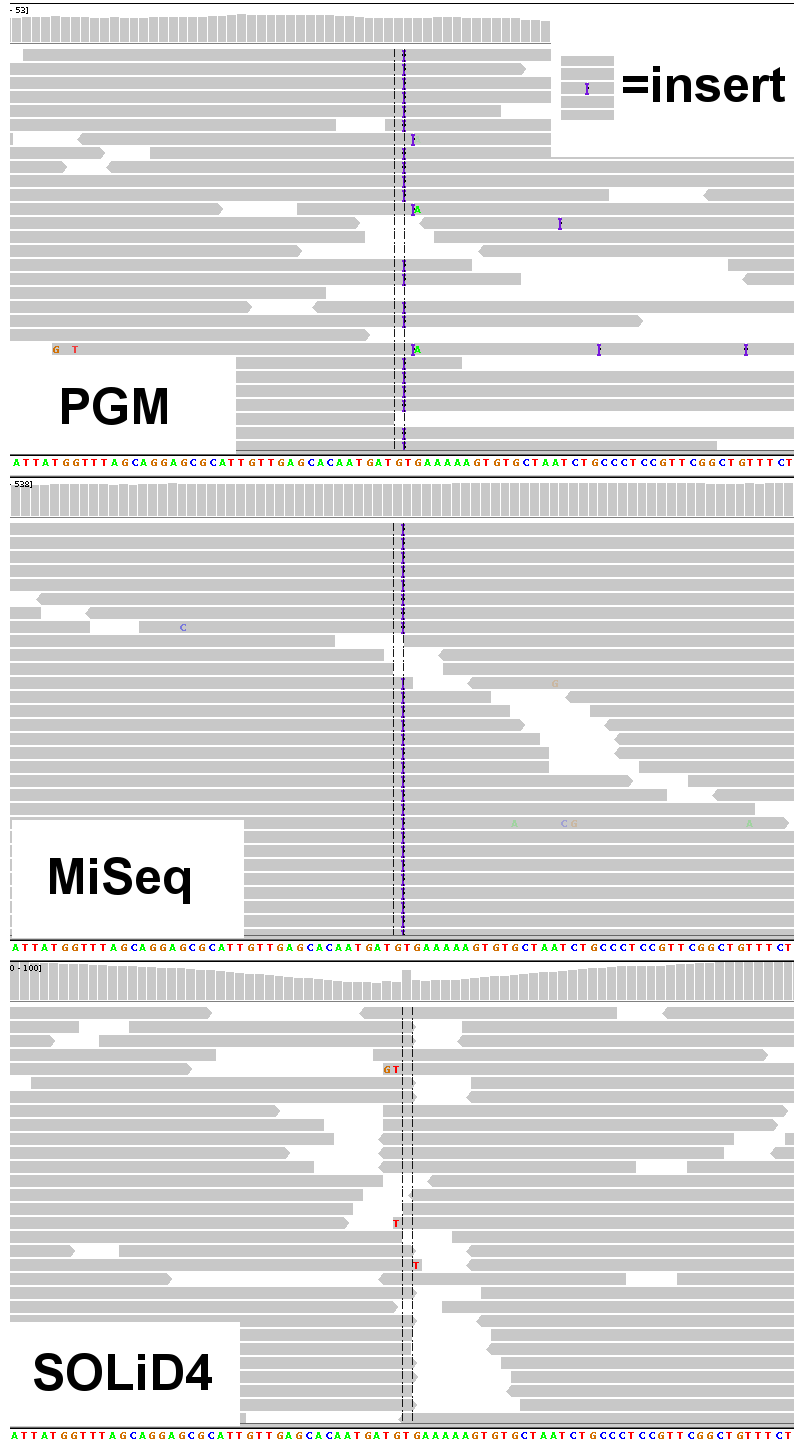

Supplement: Figure S1 — A potential novel insertion detected in DH10B. The top, middle and bottom are IGV pileups at a selected region generated by data from MiSeq, filtered PGMTM, and SOLiD 4 respectively. The selected region showed a potential novel insertion that was not found in the DH10B genome (highlighted by dotted lines). The insertion was not detected in SOLiD4 possibly because the position of the insertion was mainly covered at the end of reads. (TIF) [file pone.0045798.s001.tif]

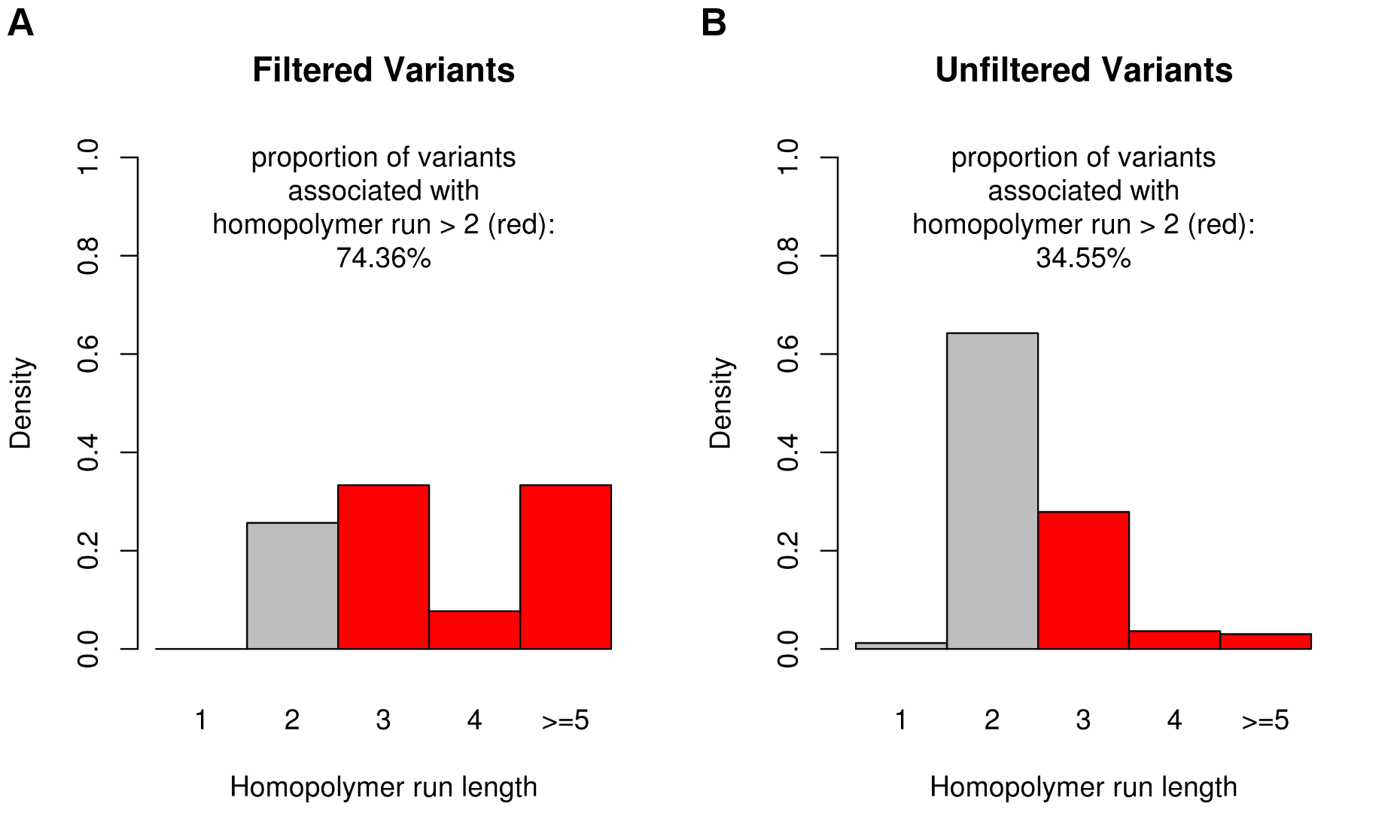

Supplement: Figure S2 — Homopolymer run lengths of indels filtered and unfiltered by VARW threshold in DH10B. Panels (A) and (B) are barplots showing the proportions of indels with different homopolymer run lengths in filtered and unfiltered data derived from the E.coli DH10B strain respectively. BAFth was set to zero so that only the effect of VARW was evaluated. (A) More than 74% of the filtered indels were found to be associated with homopolymer run length >2 bases; (B) In contrast, only about 34.55% were mapped to the same homopolymer run length profile. (TIF) [file pone.0045798.s002.tif]

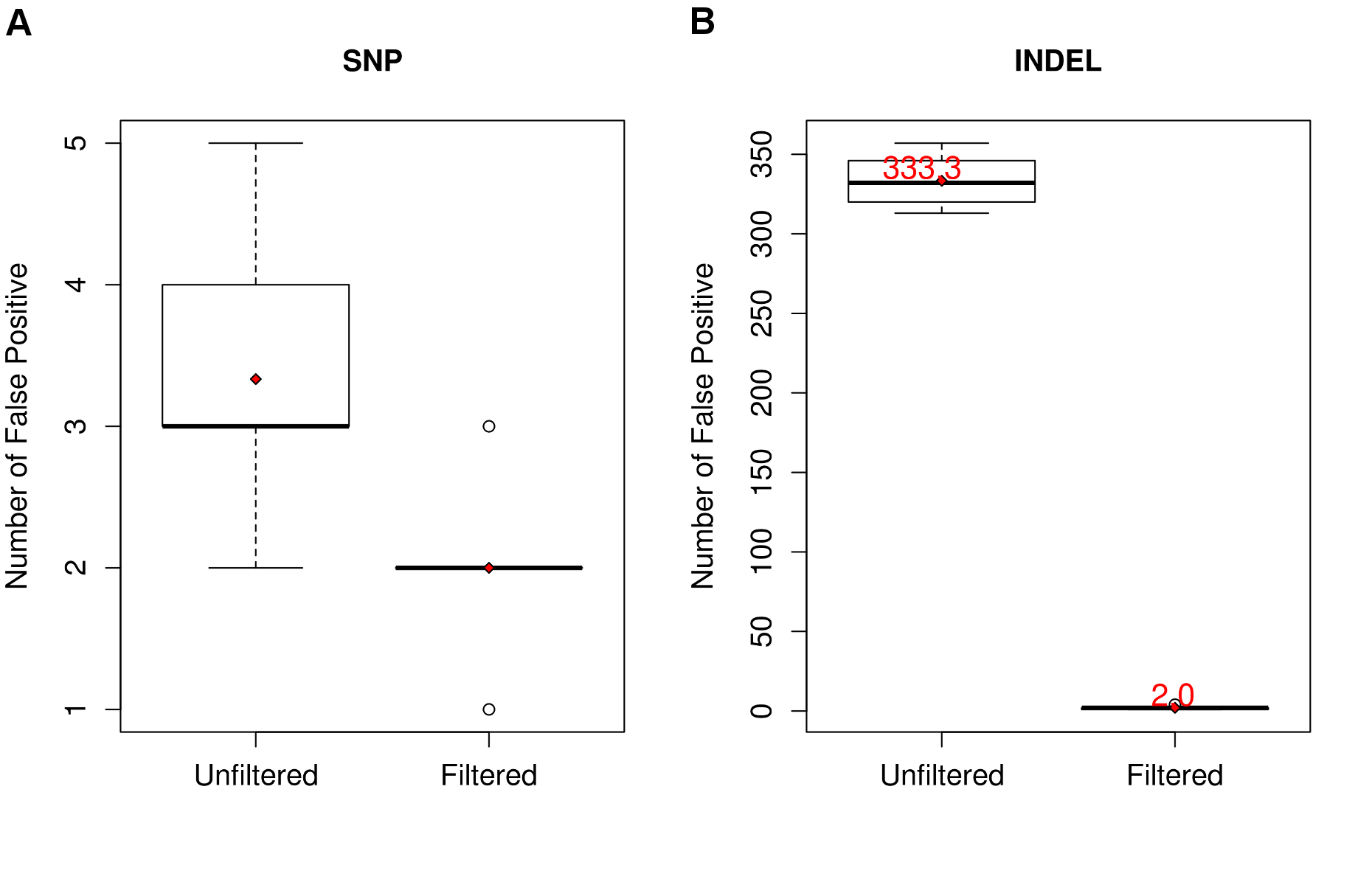

Supplement: Figure S3 — Comparison of false positive detection before and after filtering analysis. Boxplots comparing the number of false positive SNVs and indels from unfiltered and filtered results detected in the BRCA1 and BRCA2 sequences of 6 samples were plotted in (A) and (B) respectively. (A) There were less than 5 false positive SNVs detected in each of the 6 samples. (B) The red numeric numbers indicate the mean number of false positive indels before and after filtering with BAFth and VARWth. (TIF) [file pone.0045798.s003.tif]

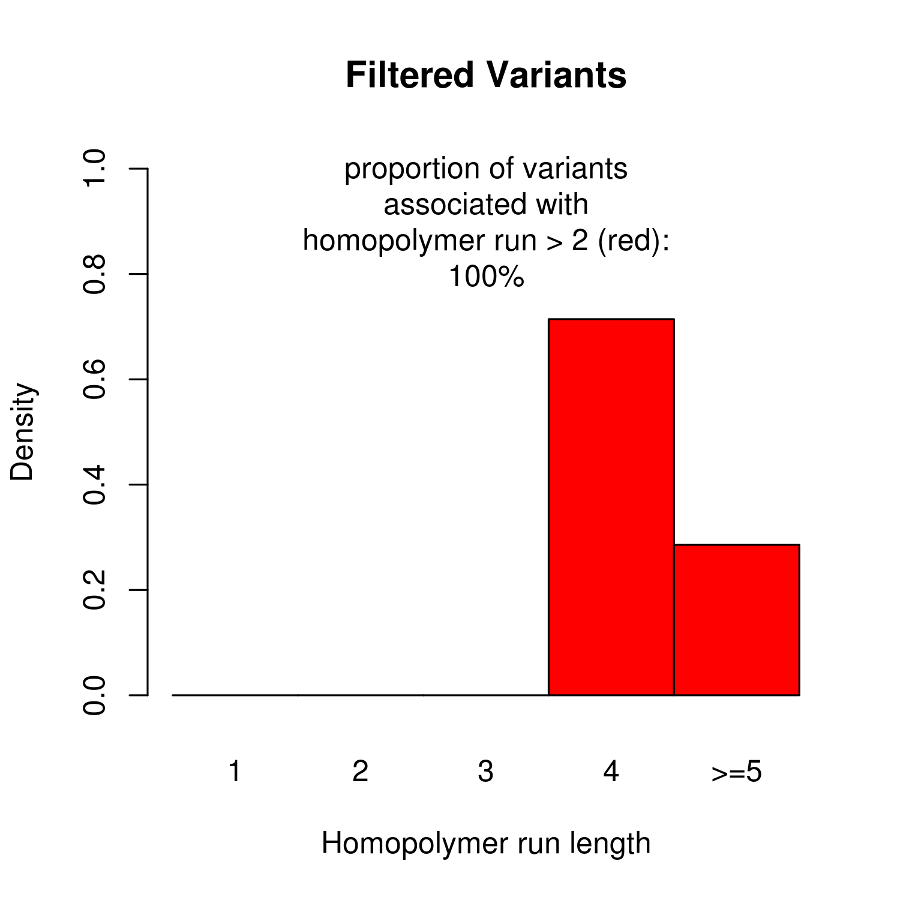

Supplement: Figure S4 — Homopolymer run length of indel errors removed only by VARW in BRCA sequences. Barplot showing the proportions of indels with different homopolymer run lengths in filtered data derived from BRCA sequences. All filtered indels were associated with homopolymer run length >3. (TIF) [file pone.0045798.s004.tif]

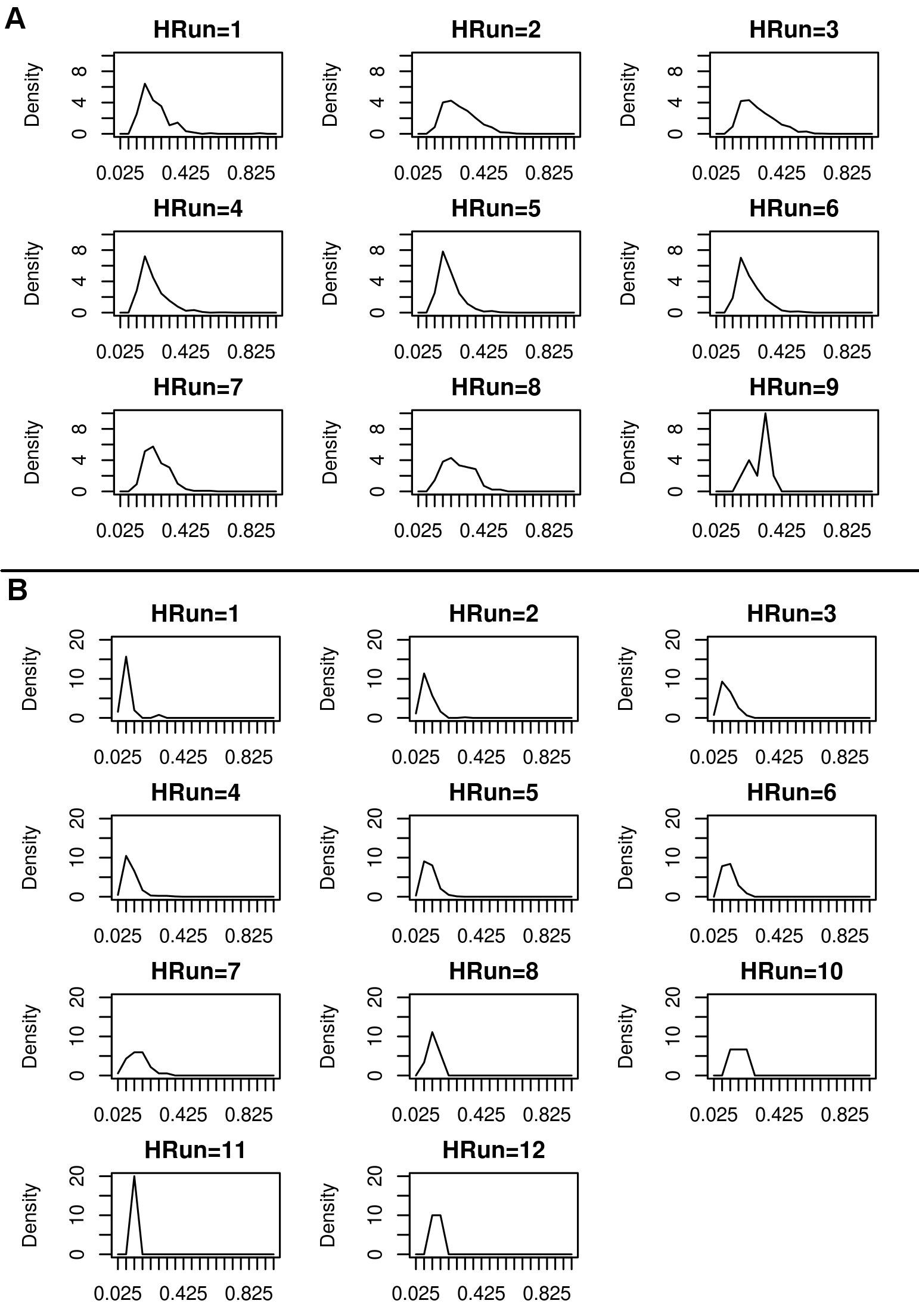

Supplement: Figure S5 — Indel BAF spectrum at different homopolymer contexts derived from DH10B and BRCA sequences. (A) Shows the indel spectrum derived from DH10B. (B) Shows the corresponding spectrum for the BRCA genes for samples S3, S5 and S6. The 'HRun' refers to the homopolymer run length which indicates that the spectrum was plotted under a given run length (homopolymer context). Samples S3, S5 and S6 were shown to have 3 indels in total by SOLiD and Sanger resequencing. The density shifted toward higher BAF values along with the increase of HRun in both (A) and (B). (TIF) [file pone.0045798.s005.tif]
